# Evaluation of WHO screening algorithm for the presumptive treatment of asymptomatic rectal gonorrhoea and chlamydia infections in at-risk MSM in Kenya

**DOI:** 10.1136/sextrans-2013-051078

**Published:** 2013-12-10

**Authors:** Eduard J Sanders, Elizabeth Wahome, Haile Selassie Okuku, Alexander N Thiong'o, Adrian D Smith, Sarah Duncan, John Mwambi, Juma Shafi, R Scott McClelland, Susan M Graham

**Affiliations:** 1 Centre for Geographic Medicine Research – Coast, Kenya Medical Research Institute (KEMRI), Kilifi, Kenya; 2 Nuffield Department of Clinical Medicine, University of Oxford, Headington, UK; 3 Department of Public Health, University of Oxford, Headington, UK; 4 The Churchill Hospital, University Hospitals, Oxford, UK; 5 University of Nairobi, Nairobi, Kenya; 6 University of Washington, Seattle, Washington, USA

**Keywords:** NEISSERIA GONORRHOEA, CHLAMYDIA TRACHOMATIS, MEN, TREATMENT, PUBLIC HEALTH

## Abstract

**Objectives:**

The WHO recommends that men who have sex with men (MSM) reporting unprotected receptive anal intercourse (RAI) and either multiple partners or a partner with a sexually transmitted infection (STI) in the past 6 months should be presumptively treated for asymptomatic rectal *Neisseria gonorrhoeae* (NG) and *Chlamydia trachomatis* (CT) infections. We evaluated this recommendation in a cohort of ‘high-risk’ MSM in Coastal Kenya.

**Methods:**

We assessed presence of genitourinary and rectal symptoms, and determined prevalence and 3-month incidence of rectal NG and CT infections. We performed nucleic acid amplification testing of urine and rectal swab samples collected from MSM followed prospectively, and assessed predictive values of the WHO algorithm at baseline screening.

**Results:**

Of 244 MSM screened, 240 (98.4%) were asymptomatic, and 147 (61.3%) reported any RAI in the past 6 months. Among 85 (35.4%) asymptomatic MSM meeting criteria for the WHO presumptive treatment (PT) recommendation, we identified 20 with rectal infections (six NG, 12 CT and two NG–CT co-infections). Among 62 asymptomatic MSM who did not meet criteria, we identified seven who were infected. The sensitivity and specificity of the WHO algorithm were 74.1% (95% CI 53.7% to 88.9%) and 45.8% (95% CI 36.7% to 55.2%), respectively. The 3-month incidence of any rectal NG or CT infection in asymptomatic men reporting any RAI was 39.7 (95% CI 24.3 to 64.8) per 100 person-years.

**Conclusions:**

About one-third of asymptomatic MSM were eligible to receive PT for NG and CT infections. Among MSM who would qualify for PT of rectal STIs, the number needed to treat in order to treat one infection was four. Our results support the value of the WHO screening algorithm and recommended PT strategy in this population.

## Introduction

Men who have sex with men (MSM) in Africa require urgent interventions to reduce acquisition and transmission of HIV-1, but focused approaches are yet to be implemented.^1^ In 2011, the WHO recommended that asymptomatic MSM reporting unprotected receptive anal intercourse (RAI) and either multiple sex partners or a sex partner with a sexually transmitted infection (STI) in the past 6 months should be presumptively treated for rectal *Neisseria gonorrhoeae* (NG) and *Chlamydia trachomatis* (CT) infections.^2^ This WHO recommendation has not been evaluated in resource-limited countries, nor has it been mentioned in WHO's renewed commitment to STI prevention and control in achieving global sexual and reproductive health.^3^ A recent study among MSM sex workers in Cote D'Ivoire, finding a high burden of rectal NG, called for validation of the WHO algorithm.^4^ We evaluated this recommendation in a cohort of MSM followed for HIV-1 and STI acquisition risks in Coastal Kenya.^5^ ^6^

## Methods

Between July and October 2011, HIV-1 negative and positive MSM in follow-up in previously described cohort studies in Coastal Kenya^5^ ^6^ were screened for NG and CT using a nucleic acid amplification test (NAAT; Gen-Probe Aptima Combo 2 assay, San Diego, California, USA).^7^ MSM were recruited for these cohort studies if they reported multiple sex partners, anal intercourse, sex work or an STI in the past 3 months. MSM who were HIV-1 negative at screening were enrolled in an open HIV-1 vaccine feasibility cohort^5^; MSM who were HIV-1 positive at screening were enrolled in a parallel cohort to receive HIV-1 care at the same research clinic.^8^ Enrolled men received quarterly risk-reduction counselling and HIV-1 testing (if previously seronegative), and had a medical history and physical examination at each scheduled visit. Face-to-face interviews were used to ascertain sexual risk behaviour and determine if WHO criteria for presumptive treatment (PT) were met. As cohort subjects had quarterly risk assessments, we used routinely collected cohort data to establish whether RAI was unprotected. For each subject, we determined whether condoms were used with the last sexual partner, with up to three identifiable partners in the previous month, and for all RAI over the previous 3-month period. For MSM who were in follow-up for 6 months or more (>75% of total), we also included data from the preceding quarterly cohort visit to establish whether RAI was unprotected over the 6-month period targeted by the WHO guidelines. At each cohort visit, men were asked if they had dysuria, urethral or rectal discharge, or rectal pain. All men submitted a urine specimen and had a rectal swab collected by a clinician.^9^ Each sample was tested for NG and CT using the Aptima Combo 2 assay. Men with rectal or urethral symptoms compatible with infection or NAAT-confirmed infections received cefixime (400 mg immediately) and doxycycline (100 mg twice a day for 7 days), risk-reduction counselling, and advice on partner treatment. Patients were given the option to take medication for their sex partner(s) or refer their partner(s) to the research clinic for treatment. Men reporting any RAI were invited for rescreening for urethral and rectal NG and CT infections at their scheduled quarterly visit.

### Data analysis

Sociodemographic and behavioural risk factors for asymptomatic (prevalent) rectal NG or CT infections were summarised for men reporting any RAI. Categorical variables were tested using χ^2^ tests. Prevalence ratios were used to measure associations between potential risk factors and baseline NG or CT infection. A multivariable exact poisson regression model was used to estimate adjusted prevalence ratios. We calculated the area under the receiver operator characteristic curve (AUC) for the predictive ability of the WHO algorithm and alternative risk criteria to identify patients with asymptomatic rectal infections for PT. The 3-month incidence rate of any rectal NG or CT acquisition was expressed as incidence per 100 person-years (PY). Cox proportional hazards models were used to assess risk factors for incident rectal NG or CT infection.

## Results

A total of 244 MSM had a urine and rectal sample collected for evaluation at baseline, of whom four (1.6%) had a symptomatic STI, including one with urethral discharge, one with dysuria, one with rectal pain, and one with rectal pain and discharge. Of the four symptomatic infections, three (75%) were NAAT-confirmed (two NG and one NG–CT co-infection, table 1). Of 240 asymptomatic men, 147 (61.2%) reported any RAI in the past 6 months, and 93 (38.8%) did not report any RAI. Overall, 28 (11.7%) of 240 asymptomatic men had an anogenital infection diagnosed. In 147 MSM reporting any RAI, 27 (18.4%) had anogenital infections, including all rectal NG infections and all but one rectal CT infections. In 93 MSM not reporting any RAI, one (1.1%) had a rectal CT infection (table 1). Upon chart review of the latter patient, RAI had been documented by the clinician, but was not admitted to in the structured risk assessment.

**Table 1:** Evaluation of the WHO screening algorithm for presumptive treatment of asymptomatic rectal gonorrhoea and chlamydia infections in 244 MSM, Coastal Kenya, 2011–2012

	Total	MSM with any rectal infection	MSM with *Chlamydia trachomatis*		MSM with *Neisseria gonorrhoea*	
			Rectum	Urethra	Rectum	Urethra
Symptomatic	4	3 (75)	–	1 (25)	3 (75)	2 (50)
Asymptomatic	240	28 (12)	20 (8)	14 (6)	11 (5)	2 (1)
No RAI	93	1 (1)	1 (1)	4 (4)	–	–
Any RAI	147	27 (18)	19 (14)	10 (7)	11 (8)	2 (1)

Values are n (%).

MSM, men who have sex with men; RAI, receptive anal intercourse.

Sociodemographic and behaviour characteristics of 147 MSM who reported any RAI, with and without rectal infections, are shown in table 2. The median age of men reporting RAI was 26 years (IQR 23–31), approximately half (47.6%) had primary or no education, the majority (89.1%) were single, three out of four men (75.5%) reported having received money or goods for sex in the past 3 months, about half (53.1%) of the men reported sex with men exclusively, and 59 (40.1%) were HIV-1-infected. Most men (73.5%) had spent more than 6 months in cohort follow-up. Age, being single, reporting multiple sex partners in the past month and reporting unprotected RAI in the past 6 months were associated with rectal infections in bivariable analysis (at p=<0.2). Notably, there were no differences in the prevalence of rectal infection between HIV-1-negative and HIV-1-positive MSM in this analysis (19/88 vs 8/59, p=0.2). No factor predicted rectal infections in multivariable poisson regression (data not shown).

**Table 2:** Characteristics of 147 MSM who reported receptive anal intercourse and factors associated with prevalent rectal *N. gonorrhoeae* (NG) or *C. trachomatis* (CT) infections, coastal Kenya, 2011–2012

Socio-demographic & behaviour characteristics	Total N=147	Rectal NG or CT infection	PR* (95% CI)	p Value
	n (%)	N=27		
Age group (years)				0.23
18-24	63 (42.9)	16 (59.3)	Referent	
25-34	70 (47.6)	9 (33.3)	0.51 (0.22–1.15)	
>34	14 (9.5)	2 (7.4)	0.56 (0.13–2.458)	
Education				0.92
None/Primary	70 (47.6)	13 (48.2)	Referent	
Secondary	57 (38.8)	11 (40.7)	1.03 (0.47–2.32)	
Higher/Tertiary	20 (13.6)	3 (11.1)	0.81 (0.23–2.83)	
Marital Status				0.09
Single	131 (89.1)	27 (100.0)	Referent	
Ever married	16 (10.9)	0 (0)	4.7 (0.83–infinity)	
Received payment for sex				0.22
No	36 (24.5)	4 (14.8)	Referent	
Yes	111 (75.5)	23 (85.2)	1.86 (0.64–5.4)	
Sexual orientation				0.3
Men only	78 (53.1)	17 (63.0)	Referent	
Men and women	69 (46.9)	10 (37.0)	0.66 (0.30–1.45)	
HIV Status				
Negative	88 (59.9)	19 (70.4)	Referent	0.26
Positive	59 (40.1)	8 (29.6)	0.63 (0.27–1.43)	
Duration of follow-up in cohort				0.74
0–1 months	22 (15.0)	5 (18.5)	Referent	
1–6 months	17 (11.6)	4 (14.8)	1.04 (0.28–3.86)	
>6	108 (73.5)	18 (66.7)	0.73 (0.27–1.98)	
**Risk factors for WHO screening for Presumptive Treatment**				
Sexual partners last month				0.17
0-1	24 (16.3)	2 (7.4)	Referent	
>1	123 (83.7)	25 (92.6)	2.44 (0.58–10.30)	
Sex partner with an STI last month				0.49
No	137 (93.2)	26 (96.3)	Referent	
Yes	10 (6.8)	1 (3.7)	0.53 (0.07–3.88)	
Unprotected RAI over the past 6 months				0.06
Yes	96 (65.3)	22 (81.5)	2.34 (0.89–6.17)	
No	51 (34.7)	5 (18.5)	Referent	
Met WHO risk criteria for PT†				0.08
Yes	85 (57.8)	20 (74.1)	2.08 (0.88–4.93)	
No	62 (42.2)	7 (25.9)	Referent	

*Prevalence ratios.

†WHO risk criteria for presumptive treatment of rectal infections (i.e. unprotected receptive anal intercourse and either multiple partners or a partner with an STI in the past 6 months.

MSM, men who have sex with men; PT, presumptive treatment; RAI, receptive anal intercourse; STI, sexually transmitted infection.

### WHO risk criteria at baseline

**Figure 1:**
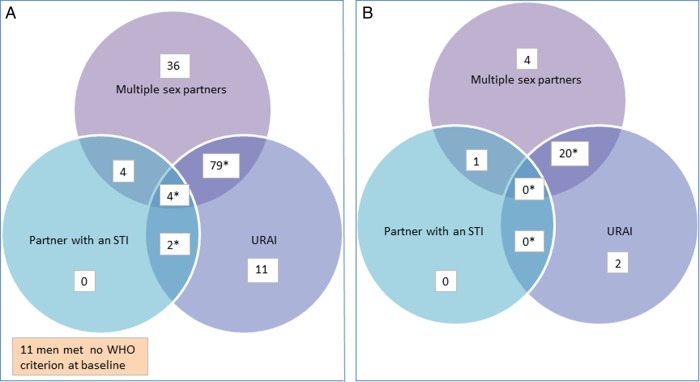
WHO criteria for presumptive treatment of *Neisseria gonorrhoeae* and *Chlamydia trachomatis* include reporting (1) unprotected receptive anal intercourse (URAI) and (2) either multiple partners or a partner with a sexually transmitted infection (STI). (A) Distribution of WHO risk criteria (ie, URAI, multiple partners and partner with an STI) among 147 men who have sex with men (MSM) reporting any RAI, Coastal Kenya, 2011–2012. (B) Distribution of WHO risk criteria (ie, URAI, multiple partners and partner with an STI) among 27 MSM with asymptomatic rectal infections, Coastal Kenya, 2011–2012. Note that none of the 11 men who met no risk criteria had an asymptomatic rectal infection.

A total of 123 (83.7%) of 147 men reporting any RAI also reported multiple sex partners in the past month; 10 (6.8%) reported a partner with an STI; and 96 (65.3%) reported unprotected RAI. A total of 85 (57.8%) MSM reporting any RAI qualified for PT because they both reported unprotected RAI and met one or both of the other two criteria (ie, 79 of the men who reported unprotected RAI also reported multiple partners, four also reported both multiple partners and a partner with an STI, and two also reported a partner with an STI; figure 1A). Eleven MSM met none of the WHO risk criteria for PT (figure 1A).

### Predictive values of WHO PT algorithm for asymptomatic rectal infections

Of all (n=240) the asymptomatic MSM, 85 (35.4%) met WHO criteria for PT, and 20 (23.5%) of these 85 MSM had a rectal infection diagnosed. Of 62 men reporting RAI who did not meet WHO criteria, seven (11.3%) had a rectal infection diagnosed. The sensitivity and specificity of the WHO algorithm for predicting rectal infection were 74.1% and 45.8%, respectively. The positive and negative predictive values of the algorithm were 23.5% and 88.7%, respectively, and the AUC was 0.60 (table 3). The predictive values of alternative risk criteria for identifying patients with asymptomatic rectal infections are shown in table 3. AUCs for these alternatives were similar to those for the WHO algorithm.

**Table 3:** Predictive values of WHO's presumptive treatment algorithm and alternative criteria for treatment of asymptomatic rectal infections in MSM, Coastal Kenya, 2011–2012

MSM (N=147) reporting RAI and meeting risk criteria in past 6 months	MSM with any rectal infection (N=27), N (%)	MSM without rectal infection (N=120), N (%)	Sensitivity, % (95% CI)	Specificity, % (95% CI)	PPV, % (95% CI)	NPV, % (95% CI)	AUC
WHO risk criteria* (N=85)	20 (23.5)†	65 (76.5)	74.1 (53.7 to 88.9)	45.8 (36.7 to 55.2)	23.5 (15.0 to 34.0)	88.7 (78.1 to 95.3)	0.60
Did not meet WHO risk criteria (N=62)	7 (11.3)‡	55 (88.7)					
Multiple partners (N=123)	25	98	92.6 (75.7 to 99.1)	18.3 (11.9 to 26.4)	20.3 (13.6 to 28.5)	91.7 (73.0 to 99.0)	0.56
No multiple partners (N=24)	2	22					
Unprotected RAI (N=96)	22	74	81.5 (62.0 to 93.7)	38.3 (29.6 to 47.7)	22.9 (15.0 to 32.6)	90.2 ( 78.6 to 96.7)	0.60
No unprotected RAI (N=51)	5	46					
Partner with STI (N=10)	1	9	3.7 (0.1 to 19.0)	92.5 (86.2 to 96.5)	10.0 (0.3 to 44.5)	81.0 ( 73.4 to 87.2)	0.52
No partner with STI (N=137)	26	111					
Multiple partners or partner with STI (N=125)	25	100	92.6 (75.7 to 99.1)	16.7 (10.5 to 24.6)	20.0 (13.4 to 28.1)	90.9 ( 70.8 to 98.9)	0.55
No multiple partners and no partner with STI (N=22)	2	20					
Multiple partners or unprotected RAI (N=136)	27	109	100.0 (87.2 to 100.0)	9.2 (4.7 to 15.8)	19.9 (13.5 to 27.6)	100.0 (71.5 to 100.0)	0.50
No multiple partners and no unprotected RAI (N=11)	0	11					
Unprotected RAI or partner with STI (N=100)	23	77	85.2 (66.3 to 95.8)	35.8 (27.3 to 45.1)	23.0 (15.2 to 32.3)	91.5 ( 79.6 to 97.6)	0.61
No unprotected RAI and no partner with STI (N=47)	4	43					

*WHO risk criteria for presumptive treatment of rectal infections (ie, unprotected RAI and either multiple partners or a partner with an STI in the past 6 months).

†Including six NG, 12 CT and two NG–CT co-infections of the rectum.

‡Including two NG, four CT and one NG–CT co-infection of the rectum.

AUC, area under receiver operating characteristic curve; MSM, men who have sex with men; NPV, negative predictive value; PPV, positive predictive value; RAI, receptive anal intercourse; STI, sexually transmitted infection.

### Incident rectal infections

Of 147 asymptomatic MSM reporting RAI at the initial visit, 128 were rescreened after a median 103 days (IQR 93–127), and 16 (12.5%) had an asymptomatic rectal NG or CT infection. Of the 16 incident infections (five NG, nine CT and two NG–CT co-infections), eight (50%) occurred in men who were treated at baseline. Eleven (69%) of the 16 infected patients met WHO criteria for PT at 3 months. Reasons for not rescreening 19 of the subjects who reported RAI at baseline included reported migration out of the study area and withdrawal from the study (n=7 and n=3, respectively), loss to follow-up (n=6), and missed opportunities for specimen collection (n=3). The incidence was 17.4 (95% CI 8.3 to 36.4) per 100 PY for rectal NG infection, 27.3 (95% CI 15.1 to 49.3) per 100 PY for rectal CT infection, and 39.7 (95% CI 24.3 to 64.8) per 100 PY for any rectal NG or CT infection. None of the characteristics presented in table 2 was a significant predictor of incident infection. However, men who had a rectal infection at baseline had a relative hazard of 8.6 (95% CI 2.9 to 25.4) for any incident rectal infection at 3 months. Men who had both a baseline and an incident infection were infected with the same organism in all cases (two NG and six CT infections). Of these eight men, only three (all with CT infections) had requested to be given treatment for their partners.

## Discussion

Over one-third of participating MSM were eligible to receive PT for NG or CT infection according to WHO criteria. The WHO algorithm was 74% sensitive for detecting rectal infections in ‘at-risk’ MSM, but had low specificity as expected. For every four MSM meeting the criteria, one infection would be treated in this population. Overall, the WHO algorithm performed poorly, and this would not improve using alternative criteria. While the WHO algorithm currently requires that healthcare workers ask men about three risk factors (ie, unprotected RAI, sex with multiple partners, and partners with an STI), a PT algorithm based only on unprotected RAI in the past 6 months would be easier to use. According to our results, such an algorithm would have a slightly better sensitivity but lower specificity. Similarly to the WHO algorithm, for every four MSM reporting unprotected RAI, one infection would be treated.

Recent studies among mostly MSM sex workers in capital cities of Uganda, Kenya and Cote d'Ivoire reported high (3.1–8.5%) rectal NG prevalence,^4^ ^10^ ^11^ but data were not presented specifically for MSM reporting RAI. In a recent study by our group, RAI and symptomatic NG infection in the past 6 months were the strongest predictors of HIV-1 acquisition in MSM, who had an overall HIV-1 incidence of 8.6 (95% CI 6.7 to 11.0) per 100 PY. As Kenyan MSM are often unaware of the risks that RAI poses for HIV-1 and STI acquisition,^12^ risk-reduction counselling for these men should focus on sexual role-taking and condom use. However, frontline health workers in Kenya lack sensitivity training on male same-sex behaviour and the prevention needs of MSM, and may find it difficult to establish who qualifies for PT or provide effective counselling messages.^1^ ^13^ They also face challenges in offering effective treatment, as national Kenyan guidelines recommend quinolones as a first-line regimen, and wide-spread resistance of NG to quinolones has emerged in Kenya.^14^ Directly observed treatment with ceftriaxone (250 mg intramuscularly immediately) or cefixime (400 mg by mouth immediately) and doxycycline (100 mg by mouth twice a week for 7 days) or azithromycin (1.0 g by mouth immediately) are currently the optimal PTs for ‘at-risk’ MSM meeting WHO criteria.

Fifty per cent of the rectal infections (n=8) we identified at 3 months were possible re-infections or treatment failures. These included two patients with an NG infection who had received directly observed treatment (but no partner treatment), and six men with a CT infection, of whom only three requested treatment for their partners. While some baseline CT infections may have been insufficiently treated because of non-completion with a 7-day course of doxycycline, it is clear that a more aggressive approach to partner treatment is required. We documented very high 3-month incidences of NG or CT infections in asymptomatic MSM reporting RAI. However, this study was too short to determine the optimal frequency of PT and was not powered to evaluate the effectiveness of PT in reducing the burden of asymptomatic STI among at-risk MSM. Offering PT to MSM reporting RAI without strong emphasis on treating recent sex partners is likely to reduce the effectiveness of a PT programme. A future evaluation of WHO's PT algorithm may be needed to determine the impact of PT plus standard partner referral for STI treatment versus PT with a more intensive approach for partner notification and treatment.^15^ In addition, ongoing surveillance is needed to monitor for drug resistance and ensure that recommended treatment regimens are efficacious.

This study has several limitations. MSM in our study often reported sex work and therefore do not represent MSM outside of the sex trade who may qualify for PT. While these men had access to prevention services through ongoing participation in a research cohort, they may have over-reported protected RAI at repeat visits out of a desire to continue in the ‘high-risk’ cohort. MSM in our study may also have been more open to report risk behaviour than can be expected at routine care services in Kenya.

In summary, we documented a high burden of rectal NG and CT infections in MSM reporting RAI who had access to STI screening services in Coastal Kenya. While the majority of MSM included in our study had regular risk-reduction counselling, the proportion of men reporting 100% condom use for all RAI episodes was low, and merits further study. These results support the value of the WHO screening algorithm and recommended PT strategy in this population. However, information on optimal frequency of PT and the overall effectiveness of a PT programme remain elusive. We recommend further evaluation of the impact of PT on the STI burden among MSM and their sexual partners, ideally in conjunction with a partner STI treatment programme.

**Key messages**

- The WHO screening algorithm for identification of at-risk men who have sex with men (MSM) for presumptive treatment had ∼74% sensitivity but low specificity for detection of asymptomatic rectal *Neisseria gonorrhoeae* (NG) and *Chlamydia trachomatis* (CT) infections.
- In this population of Kenyan MSM, only four who met WHO criteria for presumptive treatment would need to take medication to treat one asymptomatic rectal NG or CT infection.
- A strong emphasis on partner treatment is required for MSM reporting receptive anal intercourse, as 50% of the incident rectal infections at 3 months were possible re-infections.
